# Rate of Evolution in Brain-Expressed Genes in Humans and Other Primates

**DOI:** 10.1371/journal.pbio.0050013

**Published:** 2006-12-26

**Authors:** Hurng-Yi Wang, Huan-Chieh Chien, Naoki Osada, Katsuyuki Hashimoto, Sumio Sugano, Takashi Gojobori, Chen-Kung Chou, Shih-Feng Tsai, Chung-I Wu, C.-K. James Shen

**Affiliations:** 1 Institute of Molecular Biology, Academia Sinica, Taipei, Taiwan; 2 Department of Ecology and Evolution, University of Chicago, Chicago, Illinois, United States of America; 3 Division of Biomedical Research Resources, National Institute of Biomedical Innovation, Osaka, Japan; 4 Division of Genetic Resources, National Institute of Infectious Diseases, Tokyo, Japan; 5 Laboratory of Functional Genomics, Department of Medical Genome Sciences, Graduate School of Frontier Sciences, University of Tokyo, Tokyo, Japan; 6 Center of Information Biology, National Institute of Genetics, Mishima, Japan; 7 Department of Life Science, Chang Gung University, Tao-Yuan, Taiwan; 8 Division of Molecular and Genomic Medicine, National Health Research Institute, Miaoli, Taiwan; University of Dublin, Ireland

## Abstract

Brain-expressed genes are known to evolve slowly in mammals. Nevertheless, since brains of higher primates have evolved rapidly, one might expect acceleration in DNA sequence evolution in their brain-expressed genes. In this study, we carried out full-length cDNA sequencing on the brain transcriptome of an Old World monkey (OWM) and then conducted three-way comparisons among (i) mouse, OWM, and human, and (ii) OWM, chimpanzee, and human. Although brain-expressed genes indeed appear to evolve more rapidly in species with more advanced brains (apes > OWM > mouse), a similar lineage effect is observable for most other genes. The broad inclusion of genes in the reference set to represent the genomic average is therefore critical to this type of analysis. Calibrated against the genomic average, the rate of evolution among brain-expressed genes is probably lower (or at most equal) in humans than in chimpanzee and OWM. Interestingly, the trend of slow evolution in coding sequence is no less pronounced among brain-specific genes, vis-à-vis brain-expressed genes in general. The human brain may thus differ from those of our close relatives in two opposite directions: (i) faster evolution in gene expression, and (ii) a likely slowdown in the evolution of protein sequences. Possible explanations and hypotheses are discussed.

## Abstract 2

When calibrated against the genomic average, the rate of evolution among brain-expressed genes in humans is probably lower than or equal to that of other closely related primates.

## Author Summary

Whether comparing morphology or cognitive ability, it is clear that the human brain has evolved rapidly relative to that of other primates. But the extent to which genes expressed in the brain also reflect this overall pattern is unclear. To address this question, it's necessary to measure any variations in the DNA sequences of these genes between human and chimpanzee. And, to do this as accurately as possible, it's also important to require an appropriate reference group to act as a benchmark against which the differences can be measured. We therefore compared publicly available genomic sequences of chimps and humans with complementary DNA sequences of several thousand genes expressed in the brain of another closely related primate—the macaque, an Old World monkey—as well as the more distantly related mouse. Our analyses of the rates of protein evolution in these species suggest that genes expressed in the human brain have in fact slowed down in their evolution since the split between human and chimpanzee, contrary to some previously published reports. We suggest that advanced brains are driven primarily by the increasing complexity in the network of gene interactions. As a result, brain-expressed genes are constrained in their sequence evolution, although their expression levels may change rapidly.

## Introduction

The brain is a most complex and fascinating organ in mammals [1]. It has been the focal point of comparative studies among primates and mammals [2,3]. Recent developments in molecular, cellular, and cognitive methods have significantly advanced the comparative approach [4,5]. Because earlier studies have shown rapid sequence evolution in some genes in association with the rapid evolution in phenotypes [6,7], it seems natural to expect rapid molecular evolution in brain-expressed genes in humans as well.

As brain-specific genes in mammals have been shown to exhibit a lower rate of evolution than genes expressed in other tissues [8–10], the question is whether that trend is reversed in the human lineage, or whether it is accentuated in the descent of humans. The hypothesis that sequence evolution might have sped up in humans has been bolstered by the observation of rapid evolution in the expression levels of genes in the human brain [5,11]. A recent study has indeed reached such a conclusion on sequence evolution [12]. Because this study was based on comparison with a small set of “housekeeping genes,” the possibility that the control group, rather than the brain-expressed genes, deviate from the norm has to be carefully evaluated.

For comparative studies, we obtained high-quality full-length cDNAs from the brain of an Old World monkey (OWM) (a cynomolgus monkey, Macaca fascicularis) for two reasons. First, the OWM is well positioned for two types of three-way comparisons: mouse–OWM–human and OWM–human–chimpanzee. Second, high-quality brain cDNAs can be ethically obtained from OWMs under controlled conditions. We sequenced clones of full-length cDNA libraries from different regions of the brain (frontal lobe, temporal lobe, occipital lobe, and brain stem; see Protocol S1).

## Results

### Cloning and Sequencing of Brain-Expressed Genes in the Cynomolgus Monkey

In total, 60,000 cDNA clones were subjected to 5′-end sequencing, and 19,400 different transcription units were identified. Among these transcription units, 4,600 clones were randomly chosen for full-insert sequencing. The average length of these cDNAs after sequencing was 1,770 bp. Among them, 65%, or 2,996, matched RefSeq entries. The rest of the sequences matched either human expressed sequence tags or human genomic sequences. With redundancy excluded, the number of protein-coding sequences in our dataset was 2,498, with the average coding sequence (CDS) being 335 codons in length. Most of the sequences (1,774, or 75%) had more than 50% of their length in CDS. These cDNA sequences played a central role in delineating the brain-expressed genes analyzed in this report.

In this dataset, 2,170 (87%) of the cDNAs matched at least one of the functional categories, the top 19 of which are listed in Table S1. Brain appears to express more genes in the categories of intracellular protein traffic and protein targeting and localization, but fewer genes in the categories of developmental process and cell proliferation/differentiation, when compared with the genome-wide distribution. This is consistent with the known functional requirements of the mammalian brain [13].

### Brain-Expressed Genes Have Evolved More Slowly Than Other Genes in the Genome

We calculated the number of substitutions per site for synonymous sites (Ks) and nonsynonymous sites (Ka) for each brain cDNA and the genome-wide collections of CDSs of mouse and rhesus monkey *(M. mulatta)*. The averages are given in Table 1. The cDNA samples were collected from four different regions of the brain, and although the evolutionary rates varied somewhat among different regions (Table S2), the differences were not significant. Therefore, genes from different regions were pooled in the following evolutionary analyses.

**Table 1:**
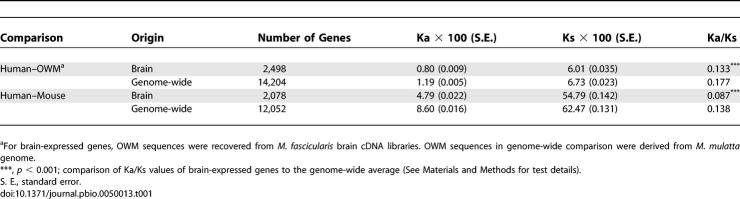
The Ka and Ks Values for Genes Expressed in the Brain Versus for All Genes in the Genome

The average Ka/Ks ratio for brain cDNAs was 0.133 and 0.087 for the human–OWM and human–mouse comparisons, respectively. We also calculated genome-wide Ka/Ks ratios for the same comparisons (0.177 and 0.138, respectively), as shown in Table 1. The Ka/Ks ratio among brain-expressed genes was significantly smaller than the genomic average (*p <* 10^−3^; see Materials and Methods). Furthermore, calculation of Ka/Ks ratio for each of 19 functional categories suggests that this slower evolution among brain-expressed genes is “global” across all functional categories (Figure S1). An exception are the genes of “electron transport,” which appear to have evolved unusually fast in the human–OWM comparison. In this category of genes, the subunits of COX and the electron transport chain components have previously been documented to be fast evolving [14–16].

**Figure 1:**
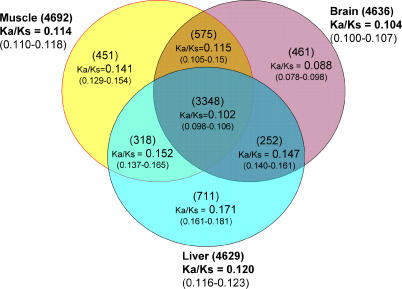
The Ka/Ks Ratios between Human and Mouse for Genes Expressed in Brain, Liver, and/or Muscle The number of genes is given in parentheses above each Ka/Ks value; the 95% confidence interval is given in parentheses below each Ka/Ks value. Expression data for brain and liver were from Enard et al. [5], and data fro muscle were from Public Expression Profiling Resource (http://pepr.cnmcresearch.org).

Another important point (the relevance of which will become clear in the Discussion) is that the slower evolution reported here was true for both brain-expressed genes and brain-specific genes, the latter being expressed exclusively or mainly in the brain. In general, tissue-specific genes evolve more rapidly than non-tissue-specific genes [10] as the latter are expected to experience broader selective constraints. This trend apparently is not true among brain-expressed genes identified by microarray hybridization experiments. In Figure 1, we compare genes expressed in any combination of brain, muscle, and liver (see Materials and Methods). Genes are classified into seven categories—those expressed in only one of the three tissues, those expressed in two of the three tissues (three categories for each), and those expressed in all three tissues (one category). Genes expressed in the brain tended to have a lower Ka/Ks, and genes that are expressed only in the brain, but not in the liver or muscle, had the lowest Ka/Ks. This observation is in agreement with Khaitovich et al.'s [17] recent study. The set of genes expressed in both brain and liver had the highest Ka/Ks among the brain-expressed gene categories, but this set was also the smallest and, hence, least statistically informative.

### Apparent Faster Evolution of Brain-Expressed Genes in Humans in the Absence of Calibration

**Figure 2:**
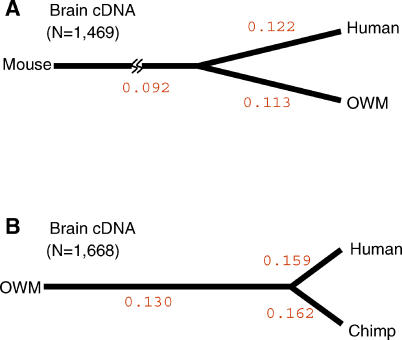
Lineage-Specific Ka/Ks Ratios for Brain-Expressed cDNAs The Ka and Ks values along each branch were calculated by the PAML method [18], and the Ka/Ks ratios are given. (A) There were 1,469 brain-expressed genes common to human, OWM, and mouse. (B) There were 1,668 brain-expressed genes common to human, chimpanzee, and OWM. For details, see Tables 2 and 3.

We next calculated the number of lineage-specific substitutions as shown in Figure 2A (human versus OWM, with mouse as the outgroup) and Figure 2B (human versus chimpanzee, with OWM as the outgroup). To separate synonymous and nonsynonymous substitutions, we used the method of Yang [18]. The rates of protein evolution among brain-expressed genes for human and OWM lineages are not significantly different (Ka/Ks = 0.122 versus 0.113; see Table 2 and Figure 2A). The rate along the mouse lineage, at 0.092, is significantly lower than those in the other two lineages (*p <* 10^−2^).

**Table 2:**
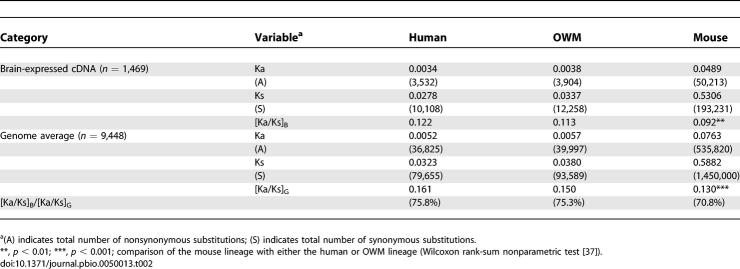
Ka and Ks Values in the Human, OWM, and Mouse Lineages as Shown in Figure 2A

**Table 3:**
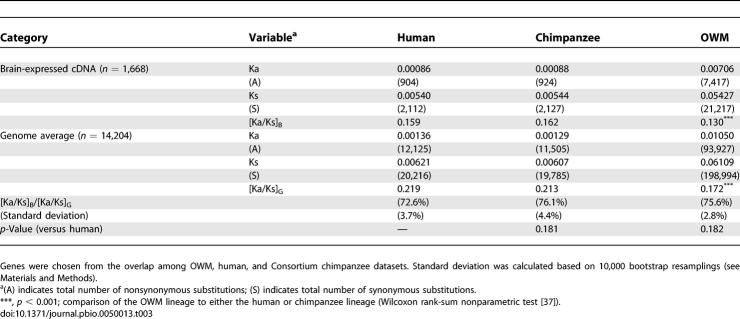
Ka and Ks Values in the Human, Chimpanzee, and OWM Lineages as Shown in Figure 2B

Because humans and OWMs have been separated from each other for more than 30 million years, changes in the last few million years in, say, the human lineage would be difficult to detect. To see the lineage effects more clearly, we used the smaller phylogeny of Figure 2B among human, chimpanzee, and OWM. In this comparison, Ka/Ks ratios in both the human and chimpanzee lineages are higher than that along the OWM lineage (about 0.160 versus 0.130; *p <* 10^−4^; see Table 3 and Figure 2B). Note that Figure 2 depicts unrooted trees and the line to the outgroup (mouse or OWM) depicts the distance from the common ancestor of human/OWM or human/chimpanzee to mouse or OWM, respectively.

Figure 2 appears to support the simple expectation, based on the phenotypic divergence of the brains, that the rate of molecular evolution follows the order apes > OWM > mouse. This conclusion is of course tentative, as it is necessary to have a proper calibration against a group of reference genes (i.e., non-brain-expressed genes). Not surprisingly, this calibration is where most studies diverge. For example, if the set of reference genes is small, one may mistakenly conclude there has been rapid evolution of brain-expressed genes when, in fact, it is the reference genes that have evolved slowly (In a previous study, Dorus et al. used only 99 slowly evolving genes for reference [12].) In what follows, we used either 4,840 or 14,204 genes as the genomic average for reference.

### Slower Evolution of Brain-Expressed Genes in Humans, Calibrated against the Genomic Average

To calibrate the distant phylogenetic comparisons of Figure 2A among human, OWM, and mouse, we used 9,488 genes for the genomic average (including 1,469 brain-expressed genes). In this set of reference genes—like for the brain-expressed genes—Ka/Ks ratios for primates were greater than that for mouse (0.161 and 0.150 versus 0.130, *p <* 10^−3^; see Table 2). Thus, the accelerated protein evolution reported in Figure 2A (see also Table 2) is a genome-wide phenomenon. When we take the Ka/Ks ratio of brain-expressed genes, [Ka/Ks]_B_, divided by that of the genome average, [Ka/Ks]_G_, the ratios are 75.8%, 75.3%, and 70.8% in the lineages of human, OWM, and mouse, respectively. Calibrated against the genome average, brain-expressed genes may indeed have evolved faster in primates than in the lineage to mouse. However, the large phylogenetic distance between rodents and primates makes this estimation less robust and less interpretable than the differences between the two primates. Between them, there is no sign of acceleration in the human lineage.

We computed the Ka/Ks ratios along the lineages to human, chimpanzee, and OWM. There are currently two large sets of chimpanzee sequences—the Chimpanzee Sequencing and Analysis Consortium (“Consortium”) sequences [19] and the Celera sequences [20]. When either dataset is used as the benchmark for the genomic average, the results agree qualitatively. The quantitative differences have turned out to be informative (see below), and they, again, illustrate the importance of calibration against the genomic average. For each dataset, we selected the chimpanzee genes that overlap with the OWM and human datasets for comparison (see Materials and Methods).

#### The Consortium sequences.

In this dataset, the genomic averages of Ka/Ks in human and chimpanzee were both higher than that in OWM (0.219 and 0.213 versus 0.172, *p <* 10^−3^; Table 3). The acceleration in the genomic average matches that in the brain-expressed genes such that the brain/genome ratios ([Ka/Ks]_B_/[Ka/Ks]_G_) are all around 75%. In this comparison, the brain/genome ratio in the human lineage (72.6%) is weakly and insignificantly (*p =* 0.18) lower than those in the other two lineages.

#### The Celera sequences.

In this dataset, the ([Ka/Ks]_B_/[Ka/Ks]_G_) ratios in the human, chimpanzee, and OWM lineages were 61.5%, 75.5%, and 90.8%, respectively (*p <* 10^−2^ in human–chimpanzee comparison), as shown in Table 4. Note that the set of brain-expressed genes is not the same as that in Table 3 because the two sets of chimpanzee sequences have different overlaps with our collection of brain-expressed genes. The slowdown in the brain/genome ratio in humans is pronounced, as seen in Table 4.

**Table 4:**
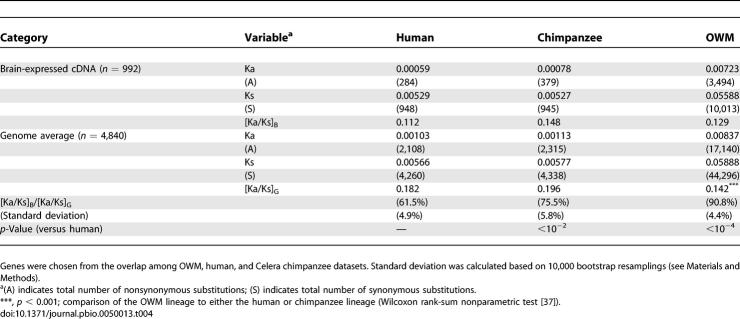
Ka and Ks Values in the Human, Chimpanzee, and OWM Lineages

#### The combined analysis of the Consortium and Celera datasets.

The differences between the two sets are largely due to gene selection, as the overlap is modest. Furthermore, for the same genes, the Celera dataset sometimes does not include all the exons. With respect to quality control, low-quality bases (quality score < 20) were masked for Consortium sequences, but quality scores are not available for Celera sequences. Nevertheless, because the Celera sequences have slightly lower Ks values, the differences cannot be attributed to possible higher error rates in that dataset.

The reason for the seemingly inconsistent results between the two datasets is due primarily to the differential inclusion of very slowly evolving genes along the human and chimpanzee lineages (Ka < 0.002). In the Celera dataset, genes with Ka = 0 are overrepresented, compared with the Consortium dataset, whereas the overrepresentation is reversed for genes with 0 < Ka < 0.002 (Figure S2). Among these genes, stochastic fluctuation is probably too large to reveal any lineage effect between human and chimpanzee. Indeed, when these very slowly evolving genes are removed from the analysis, the discrepancy disappears. In Table 5, we show the analysis of the combined Celera/Consortium datasets, consisting of genes with Ka > 0.002. The brain/genome ratios in Ka/Ks for the human, chimpanzee, and OWM lineages are, respectively, 62.9%, 69.6%, and 72.1% (Table 5).

**Table 5:**
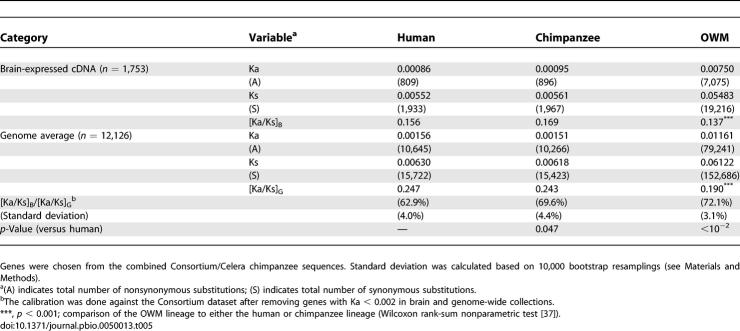
Ka and Ks Values in the Human, Chimpanzee, and OWM Lineages

In summary, calibrated against the genomic average, brain-expressed genes in humans have evolved somewhat more slowly than those in other primates. This difference appears to be more pronounced among the more conservative genes.

## Discussion

A previous report [12] on the accelerated evolution of brain-expressed genes in species with more advanced brains may have been confounded by the fact that these species tend to show an even greater acceleration among all genes in the genome. This general trend is not entirely surprising. For example, compared with rodents, primates are likely to have smaller effective population size, and selection against slightly deleterious amino acid changes would be less effective in primates [21,22]. An increase in the Ka/Ks value across the genome is thus expected. Another study of a set of 201 brain genes [17] also reported more nonsynonymous substitutions in the human lineage than in the chimpanzee lineage, although the difference was not significant and was not calibrated against synonymous changes.

What then may account for the slow sequence evolution among brain-expressed genes, when calibrated against the genomic average, in species with more advanced brains? We shall first discuss potential biases in the observations, then we will address this question.

First, our sampling might be biased toward more abundantly transcribed genes; such highly expressed genes may be slowly evolving [10]. We used two different sources of expression studies to correlate the Ka/Ks ratios of genes and their expression levels. In both cases, the correlation coefficient was very low, with *R* ^2^ ≤ 0.01 (see Protocol S1 for details). Furthermore, in the comparisons shown in Figure 1, genes were chosen from brain, liver, and muscle by the same expression criteria, and yet brain-expressed genes were still the most slowly evolving ones.

Second, sampling of brain-expressed genes could have been biased toward certain categories of genes, which may happen to be slowly evolving. For example, housekeeping genes, which generally evolve slowly, might be disproportionately represented in our collection. In Figure S1, the slowdown was observed across all known categories of genes, thus alleviating this concern.

Another potential concern is the differences between tissue-expressed and tissue-specific genes. One might argue that the accelerated morphological changes in the human brain could be correlated with the accelerated evolution of only brain-specific (but not all brain-expressed) genes. Positive selection probably works most effectively on tissue-specific genes, as there would be fewer conflicting requirements among tissues to meet. Indeed, positive selection probably accounts for the rapid evolution of genes specifically expressed in male reproductive tissues [7,23]. However, brains appear to be different, as brain-specific genes evolved even more slowly than brain-expressed genes between human and mouse (see Figure 1). We also analyzed 262 brain-specific genes in primates (from http://www.hugeindex.org) [8]. As shown in Table S3, brain-specific genes evolved even more slowly than brain-expressed genes in each of the lineages to macaque, chimpanzee, and human.

When tissue-specific genes evolve more slowly than the rest of the genome, the explanation is most likely a stronger selective constraint in that tissue. In such a case, all genes expressed in that tissue should experience some of those constraints. Brain-expressed and brain-specific genes may all be slowly evolving for reasons of shared constraints. It seems possible that the stronger selective pressure on brain-expressed genes is the consequence of the higher complexity of the biochemical network in the brain [9]. Studies from yeast and nematode also showed that proteins with more interacting partners evolved more slowly ([24,25], but see [26]). The increase in selective pressure could result from a more diverse biochemical environment of the brain. Mutations would not be tolerated if they disrupted the existing interactions [27,28].

While human cerebral cortex is severalfold larger than that of chimpanzee, it contains only 50% more neurons [1,29]. As RNA abundance is relatively constant per gram of brain tissue [29], transcript abundance per neuron must be greater in the human than in the chimpanzee brain. Moreover, in microarray comparisons, extensive expression upregulation in the human brain was observed [5,11]. On the basis of individual neurons of the brain, humans may indeed have a more active, or even more complex, transcription profile than chimpanzees.

We suggest that such abundant and complex transcription may increase gene–gene interactions and constrain CDS evolution. Can this constraint of complex interactions then be compatible with the apparent rapid evolution in the expression of brain genes in humans [29]? The mechanistic basis for protein–protein interaction driving protein sequence evolution may be quite different from that of protein–DNA interaction driving expression profile differentiation. Guss et al. [30] presented a case where a change in the protein sequence of a selector gene could change the expression of the entire regulatory network. This type of large-scale trans-regulation may be a characteristic of brain-expressed genes. In Protocol S1, we compare the level of nucleotide changes associated with expression differences in genes. Table S4 suggests that brain-expressed genes may be under more extensive trans-regulation than their liver counterparts.

Our results do not contradict the finding of accelerated amino acid changes in some brain-expressed genes [6,31,32]. Deviations from the general trend are expected. In fact, these deviations may be the most interesting cases. It has often been postulated that evolution in regulatory signals, rather than in CDSs, characterizes species divergence [33]. While the generality of this postulate remains unproven [34], the evolution of the human brain may be a relevant example.

## Materials and Methods

Details on the construction of 5′-end oligo-capped brain cDNA libraries, clone sorting and selection, and cDNA sequencing are given in Protocol S1.

### Microarray analysis.

The CEL files of brain and liver were obtained from Enard et al. [5] (http://email.eva.mpg.de/~khaitovi/supplement1.html). The CEL files of human muscle were obtained from Public Expression Profiling Resource (http://pepr.cnmcresearch.org/browse.do?action=list_prj_exp&projectId=132). Presence or absence of genes on arrays was evaluated at *p* = 0.05 using the Affymetrix PMMM analysis (http://www.affymetrix.com). A gene was defined as “expressed” in a particular tissue if it was “present” in at least half of the hybridizations. The mouse homologs of genes expressed in human brain, liver, and muscle were downloaded from ENSEMBL (http://www.ensembl.org). The 95% confidence intervals of Ka/Ks ratios were estimated by the bootstrap method. Numbers of genes resampled are based on the size of each of seven categories (Figure 1); 10,000 resamplings with replacement were performed, and Ka/Ks ratios were estimated.

### Sequence analysis.

Human and mouse CDSs were downloaded from ENSEMBL (Ensembl 35). We used two sets of chimpanzee sequences in this study. The chimpanzee genome sequences were from the Consortium dataset (http://hgdownload.cse.ucsc.edu/downloads.html) [19], and the 7,665 chimpanzee CDSs were from the Celera dataset (“Supporting Online Material” of [20], available at http://www.sciencemag.org/cgi/content/full/302/5652/1960/DC1). Genome sequence of rhesus monkey *(M. mulatta)* was downloaded from the Human Genome Sequencing Center (http://www.hgsc.bcm.tmc.edu). Genome data from rhesus monkey were used instead of genome data from cynomolgus monkey *(M. fascicularis)* simply because there are no genome data available for the latter. Nevertheless, both species belong to the OWM group and should be equal distance from apes. The CDSs of chimpanzee (Consortium) and rhesus monkey were then extracted from the genome sequences based on the annotation downloaded from http://hgdownload.cse.ucsc.edu/downloads.html. Bases with quality score < 20 were masked for further analysis. For genes with multiple transcripts, only the longest transcript was retained and used.

For the pairwise comparisons in Table 1, human and mouse CDSs were cross-blasted in blastp searches. The best hits from both blast results were treated as orthologs. Human and OWM *(M. mulatta)* CDSs were also cross-blasted, but using blastn instead. OWM CDSs with non-triplet indels were excluded (1,096 CDSs) from further analyses as their ability to be transcribed/translated is not certain. In total, 12,052 human/mouse and 14,204 human/OWM orthologous pairs were identified and served as genome-wide comparisons. Next, brain cDNAs of M. fascicularis were used as the template to blast the non-redundant (NR) NCBI database (http://www.ncbi.nlm.nih.gov/BLAST). We chose the putative orthologs of human that had the highest score and lowest *E* value in the blast search. The CDSs of M. fascicularis were then extracted based on the annotation of human CDSs. Of 2,498 putative human/M. fascicularis orthologous pairs, 2,077 of them had identifiable mouse homologs based on human/mouse cross-blast search as described above. These gene pairs were considered brain-expressed genes.

The OWM sequences used in three-species comparisons (human–OWM–mouse and human–chimpanzee–OWM) were from rhesus monkey *(M. mulatta),* and brain-expressed genes were identified from putative *M. fascicularis/M. mulatta* ortholog pairs. To construct the alignment of human–OWM *(M. mulatta)*–mouse trios, the CDSs of putative orthologs from the three species were translated and aligned using ClustalW [35], and back-translated to their corresponding DNA sequences using TRANSALIGN software from the EMBOSS package [36]. Of 9,448 aligned human–OWM–mouse trios, 1,469 were putative orthologs of brain cDNAs derived from *M. fascicularis;* these were treated as brain-expressed genes.

For human–chimpanzee (Consortium)–OWM trios, Consortium chimpanzee CDSs were cross-blasted with human and OWM CDSs in blastn searches. Only the genes consistently showing the highest score and lowest *E* value in all three-way searches (human–OWM, chimpanzee–OWM, and human–chimpanzee) were retained as putative orthologs. Human–chimpanzee (Consortium)–OWM trios were aligned as described above. In total, 14,204 genes were aligned, including 1,668 brain-expressed genes. For human–chimpanzee (Celera)–OWM trios, chimpanzee CDSs derived from Clark et al. [20] were used. The gaps in Celera chimpanzee sequences were masked with “N” and aligned with human and OWM sequences. Total numbers of aligned and brain-expressed genes in this dataset were 4,840 and 992, respectively.

We calculated the Ka and Ks values for each putative pair of orthologs using PAML [18]. Ka and Ks were estimated jointly for each ortholog using codeml with the F3x4 codon frequency model and M1 model for variable ω based on the unrooted tree. For a set of genes, Ka/Ks was calculated by summing the number of substitutions and the number of sites to obtain Ka and Ks for the concatenated set before taking the ratio.

### Statistics.

To test whether the substitution rates are significantly different between any two sets of genes, we applied the log-linear model with the quassipoisson family and default log link function. The model formula is as follows:

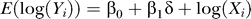

*Y_i_* and *X_i_* denote the number of substitutions and sites for each gene, respectively. In the model, log (*X_i_*) is usually called offset. δ is the indication function; δ = 1 if a gene is brain expressed, and δ = 0 if it is not. The null hypothesis is that β_1_ is not significantly different from zero. Using this model, we tested Ka/Ks for the brain-expressed genes versus the genome-wide collection (Table 1) and divergence of promoter regions for brain- versus liver-expressed genes (Table S4). Genes recovered from different brain regions were compared by the same method (Table S2).

To test the differences in [Ka/Ks]_B_/[Ka/Ks]_G_ between human and chimpanzee, we resampled equal numbers of genes from the genomes. For example, 1,668 and 1,753 genes were resampled for Tables 3 and 5, respectively, from 14,204 and 12,126 genes collected genome-wide, respectively. For Table 4,992 genes were randomly picked from the 4,840 genes. The average Ka/Ks values of the resampled data were calculated and divided by [Ka/Ks]_G_ for each of 10,000 resamplings. The mean and standard deviation of [Ka/Ks]_resampled_/[Ka/Ks]_G_ were calculated for both human and chimpanzee, and the percentages of their differences were compared with the observed differences between the two species.

The Wilcoxon rank-sum nonparametric test [37] was applied to determine whether the average Ka/Ks ratios are significantly different between lineages.

## Supporting Information

Figure S1 The Ka/Ks Ratios for Genes in Different Functional Categories The average Ka/Ks ratios of brain-expressed genes belonging in each of the 19 functional categories listed in Table 1 were calculated for the human–OWM and human–mouse comparisons. Genes from the whole genome comparison between human and mouse were adopted from Waterston et al. [38]. Note that the differences are consistent across all functional categories.
(712 KB DOC)

Figure S2 Distribution of Ka in the Lineages Leading to Great Apes (Human + Chimpanzee) for Brain-Expressed Genes in Consortium and Celera Datasets (27 KB DOC)

Protocol S1 Supplementary Materials and Methods (69 KB DOC)

Table S1 Functional Categorization of the 2,498 Protein-Coding Genes Expressed in the Brain of OWM The 2,498 genes expressed in the OWM brain are categorized according to their functions using the Panther system [39]. Only the top 19 categories in transcript abundance are shown. The percentage in parentheses is the ratio of the number of brain-expressed genes to that of all genes belonging to the same category.
(37 KB DOC)

Table S2 Ka and Ks Values for Genes Expressed in Different Regions of the Brain (31 KB DOC)

Table S3 Ka and Ks Values of Brain-Specific Genes in the Human, Chimpanzee, and OWM Lineages as Shown in Figure 2B Information on tissue specificity was originally from http://www.hugeindex.org and was used in a previous study [8]. In the present study, our sequenced brain cDNAs cover only 262 brain-specific genes.
(27 KB DOC)

Table S4 Level of Nucleotide Divergence (in Percent) in the 5′-Region of Genes with Greater than 20% or 40% Expression Difference between Human and Chimpanzee The average 5′-region divergence for all genes in the genome is given for comparison.
(26 KB DOC)

### Accession Numbers

The sequences used in this paper can be accessed via the DNA Data Bank of Japan (http://www.ddbj.nig.ac.jp) under accession number AB170063–174733.

## Abbreviations

CDS: coding sequence
Consortium: Chimpanzee Sequencing and Analysis Consortium
Ka: number of substitutions per site for nonsynonymous sites
Ks: number of substitutions per site for synonymous sites
OWM: Old World monkey
