# Supplementary material for: Rate of Evolution in Brain-Expressed Genes in Humans and Other Primates
Source: PLoS Biol. 2006 Dec 26;5(2):e13. doi: 10.1371/journal.pbio.0050013 (PMC1717015; doi:10.1371/journal.pbio.0050013)
Supplement: Supplementary file 1 [file pbio.0050013.sd001.doc]

**Supplementary Materials and Methods**

**cDNA library construction and sequencing**

Constructing libraries of 5'-end oligo-capped full-length cDNAs

cDNA libraries were constructed from cynomolgus monkey brain tissues. These cDNAs were capped with oligonucleotdies on their 5'-ends in order to preserve the full length of the transcripts[1,2]. Each clone was named according to the region of the brain from which it was collected: Qbs (brain stem), Qfl (frontal lobe), Qor (occipital lobe), Qtr (temporal lobe), Qnp(parietal lobe), Qcc (cerebellum cortex), Qmo (medulla oblongata). In this study, Qbs and Qmo clones were combined as brain stem and Qcc and Qnp clones were reserved for other broad analysis.

Properties of the oligo-capped clones

The comparison between the length distribution of macaque cDNA inserts and those of known mouse genes suggests that the 5’-capped clones are proper representation of the transcriptome. Suzuki et al.[1] analyzed 5000 cDNA clones that have good matches with known human genes at the 5'-end. Among them, 59% (approximately 3000 cDNAs) were scored as full-length. A cDNA was scored as full-length if it has the same 5’-end as, or a longer 5’-end than, any other previously reported cDNA sequence in addition to other features of the 5’-end. Because the macaque mRNAs were collected under controlled circumstances, we expected the quality of the macaque clones to exceed that of the human clones. We indeed found 70% of the macaque cDNA clones to be full length by the above criterion. However, among the clones that have the proper 5’-end, many are not complete in the 3’-end. As a result, only about 50% of the clones are full-length as determined from both ends.

Clone sorting by 5'-end sequencing

The 5’-end sequences of the clones were determined on an ABI 3700. About 10,000 clones for each library were isolated and the 5’-ends of these clones were sequenced. In total, we obtained 60,000 5’-end sequences, which resulted in 19,400 clusters. Each sequence was clustered and categorized by using DYNACLUST (DYNACOM). To determine whether they have homologous sequences (either gene or EST) in the public database, we used the BLAST algorithm. About 70% of the clones contain the initiator ATG in their sequences[1,2]. The rest do not contain the true 5’-end and may represent truncated transcripts. Clones showing homology to known gene sequences with the start codon (ATG) and an open reading frame were then chosen for further full-insert sequencing. Selection of clones for further sequencing is unbiased with respect to possible protein functions or motifs. Out of the 19,400 clusters of clones, we eventually sequenced 4,600 clones in full (see below), which represented about 20%-40% of the clones from each library that met the criteria for further sequencing.

DNA sequencing

Insert sequences of the cDNA clones were determined by the primer-walking method. The first sequencing reaction was carried out using the primer of the vector (ME-FW2: AGT-GGA-TGT-CTT-TAC-TTC-TAG-GC). After that, the primers were designed by the primer3 software[3], or manually on the basis of the obtained sequences. All the sequencing reactions were accomplished on ABI 3700 and 3100 automated sequencers (Perkin-Elmer). Cycle sequencing was done with ABI PRISM BigDye terminator Sequencing Kit (Perkin-Elmer). Primer synthesis was carried out using ABI prizm 3900. The cDNA-insert was assembled and the vector sequence removed by the DNAStar package (Lasergene).

**Functional categories of brain cDNA**

Annotation of these genes appears to reflect the putative characteristics of brain functions. We categorized the 2,498 monkey brain cDNAs by function, using the Panther classification system[4] (http://panther.celera.com). In total, 2,170 (87%) of the cDNAs matched at least one of the functional categories, the top 19 of which are listed in Table S1. We also compared our current monkey brain cDNA database to the whole-genome human transcripts from Ensembl (http://www.ensembl.org). For the latter, a total of 14,285 Ensembl human genes could be assigned to at least one of the 19 categories of Table S1. The monkey brain cDNAs thus account for 13% (1,857/14,285) of the genome-wide transcripts belonging in these categories. As shown in Table S1, brain appears to express more genes in the categories of intracellular protein traffic (24.1%), protein targeting and localization (21.1%), amino acid metabolism (17.9%), protein metabolism and modification (17.6%), and neuronal activities (16.6%) when compared with the genome-wide distribution. This is consistent with the known functional requirements of the mammalian brain. For example, most neuronal proteins are synthesized in the cell body and transported some considerable distance to the dendrites, a process requiring the coordination of intracellular protein traffic, protein targeting, and localization[5]. On the other hand, cell division and differentiation have stopped in most brain regions of the adult mammals. This is reflected in the relatively small number of expressed brain genes in the categories of developmental process (6.5%) and cell proliferation/differentiation (8.5%), respectively.

Confirming the low rate of evolution in brain-expressed genes, we now ask whether a subset of categories may account for the overall low rate, or whether the slowdown is “global”, observable across the board. A “global” low rate assures the robustness of the conclusion, as the results are less likely to be influenced by unknown biases in gene sampling. As shown in Fig. 1, the slower evolution of the mammalian brain-expressed genes can indeed be observed across all functional categories. The genes of certain categories, e.g. immunity and defense, lipid/fatty acid/steroid metabolism, cell adhesion, apoptosis, and electron transport exhibit relatively higher rates of changes. In contrast, genes belonging in other categories, such as cell structure and motility, neuronal activities and protein targeting and localization tend to be subjected to stronger functional constrain. Nevertheless, between human and mouse, the Ka/Ks ratios for brain-expressed genes are lower than those of the whole genome control in nearly all categories (the two lower profiles of Fig. S1). The profile of human/OWM (the top profile of Fig. S1) parallels closely the other two in general trends. An exception is the genes of "electron transport" which appear to have evolved unusually fast in the human/OWM comparison. In support of this interpretation, the subunits of COX and the electron transport chain components have previously been documented to be fast evolving [6-8].

**Analysis of correlation between level of expression and divergence**

We estimated the expression levels of human homologs of OWM cDNAs from two resources. First, for each gene in our dataset, numbers of human ESTs were counted from UniGene Build #162. The distribution of the EST numbers should reflect the global expression pattern of the genes. EST information could be found in NCBI for 2,174 of the 2,498 genes, with the human EST numbers ranging from 15 to 30,000 and a median of 242. Since those EST numbers are not normally distributed, the numbers were log-transformed making the data close to normal distribution. The correlation between the log-transformed EST numbers and the amino acid changes (Ka) is quite small, with R^2^ = 0.01. Furthermore, the average Ka/Ks values for genes in the top 10% and bottom 10% of the EST number are similar, at 0.15 and 0.18, respectively. Second, the homologous of 1,090 of the macaque brain genes sequenced in our study were included on the Affymetrix chips used by Enard et al.[9]. For these genes, the correlation between the expression levels estimated in that study and the Ka values is again low, with R^2^ = 0.01. Thus, the observed slowdown in evolution among the brain-expressed genes could not be accounted for by their expression levels.

**Divergence of upstream regions for brain and liver expressed genes**

To study the relationship between expressional change and regulatory region divergence, we downloaded the 1kb upstream region from the transcriptional start site of human and chimpanzee orthologs from ENSEMBL. It notes worthy that our definition potential regulatory region is arbitrary. Although the beginning of ENSEMBL transcripts is likely a transcriptional start sites, we do not know whether it is the same in liver and brain. In the promoter regions being studied, the most conserved segment is around 1kb upstream of annotated transcription start sites (Fig. 2 of [10]). Thus, we choose 1kb upstream as the cut off. Sequences which cannot be credible aligned or have divergence greater than 10% were removed from further analysis. Liver and brain expressions were originally from Enard et al[9] but were reanalyzed by Hsieh et al [11]. Genes were sorted according to their expressional differences and the cut-off was set at log2 > 0.26 (~ 20% difference) and log2 > 0.5 (~ 40% difference).

The average divergence between human and chimpanzee for 7,802 genes is 1.12% in the upstream region, which represents the genomic average. For the subset of genes with a 20% expression difference between human and chimpanzee, the level of sequence divergence for liver-expressed genes is 1.20% (P < 10^-3^, vs. the genomic average) whereas the level is 1.13% for brain-expressed genes (P >0.4; see Table S4). The same is true for genes with a 40% expression difference (Table S4). It thus appears that the evolution in expression regulation does not follow the same rule for brain- and liver-expressed genes. Perhaps in a complex biochemical environment like brain, the level of expression may change rapidly by trans-regulatory mechanisms, whereas cis-regulatory changes may often be involved in tissues with a less complex transcription profile.
