# Supplementary material for: Rate of Evolution in Brain-Expressed Genes in Humans and Other Primates
Source: PLoS Biol. 2006 Dec 26;5(2):e13. doi: 10.1371/journal.pbio.0050013 (PMC1717015; doi:10.1371/journal.pbio.0050013)
Supplement: Supplementary file 2 [file pbio.0050013.sg001.doc]

Fig. S1. The Ka/Ks ratios for genes in different functional categories.

The average Ka/Ks ratios of brain-expressed genes belonging in each of the 19 functional categories listed in Table1 were calculated for the human-OWM and human-mouse comparisons. Genes from the whole genome comparison between human and mouse were adopted from Waterston et al. [39]. Note that the differences are consistent across all functional categories.
