# Supplementary material for: Rate of Evolution in Brain-Expressed Genes in Humans and Other Primates
Source: PLoS Biol. 2006 Dec 26;5(2):e13. doi: 10.1371/journal.pbio.0050013 (PMC1717015; doi:10.1371/journal.pbio.0050013)
Supplement: Supplementary file 3 [file pbio.0050013.sg002.doc]

Fig S2 The distribution of Ka in the lineages leading to great apes (human + chimpanzee) for brain expressed genes in consortium (blue) and Celera (red) datasets.
