# Supplementary material for: Rate of Evolution in Brain-Expressed Genes in Humans and Other Primates
Source: PLoS Biol. 2006 Dec 26;5(2):e13. doi: 10.1371/journal.pbio.0050013 (PMC1717015; doi:10.1371/journal.pbio.0050013)
Supplement: Supplementary file 4 [file pbio.0050013.st001.doc]

Table S1. Functional categorization of the 2,498 protein-coding genes expressed in the brain of Old World monkey

| Functional categories | Number of matched clones  (# in our sample/  # in the genome) | | |
| --- | --- | --- | --- |
| intracellular protein traffic | 149 | | (24.1%) |
| protein targeting and localization | 27 | | (21.1%) |
| amino acid metabolism | 35 | (17.9%) | |
| protein metabolism and modification | 310 | (17.6%) | |
| neuronal activities | 58 | (16.6%) | |
| apoptosis | 43 | (14.9%) | |
| nucleoside, nucleotide and nucleic acid metabolism | 299 | (14.4%) | |
| cell cycle | 70 | (14.1%) | |
| cell structure and motility | 81 | (13.9%) | |
| oncogenesis | 45 | (13.6%) | |
| lipid, fatty acid and steroid metabolism | 71 | (13.2%) | |
| carbohydrate metabolism | 51 | (12.3%) | |
| signal transduction | 270 | (10.8%) | |
| immunity and defense | 79 | (10.1%) | |
| cell adhesion | 49 | (9.8%) | |
| Transport | 92 | (9.2%) | |
| electron transport | 19 | (9.0%) | |
| cell proliferation and differentiation | 29 | (8.5%) | |
| developmental processes | 80 | (6.5%) | |
| Total | 1857 | | (13%) |

The 2,498 genes expressed in the OWM brain are categorized according to their functions using the Panther system [38]. Only the top 19 categories in transcript abundance are shown. The percentage in the parentheses is the ratio of the number of brain-expressed genes relative to that of all genes belonging to the same category.
