# Supplementary material for: Rate of Evolution in Brain-Expressed Genes in Humans and Other Primates
Source: PLoS Biol. 2006 Dec 26;5(2):e13. doi: 10.1371/journal.pbio.0050013 (PMC1717015; doi:10.1371/journal.pbio.0050013)
Supplement: Supplementary file 5 [file pbio.0050013.st002.doc]

Table S2. The Ka and Ks values for genes expressed in different regions of the brain

| Brain Regions | Human-OWM | | | | Human-Mouse | | | |
| --- | --- | --- | --- | --- | --- | --- | --- | --- |
|  | Orthologs | Ka^A^  (S.E.) | Ks  (S.E.) | **Ka/Ks** | Orthologs | Ka  (S.E.) | Ks  (S.E.) | **Ka/Ks** |
| Brain Stem | 943 | 0.7  (0.012) | 5.8  (0.053) | **0.121** | 777 | 4.4  (0.04) | 53.2  (0.297) | **0.083** |
| Frontal Lobe | 1002 | 0.9  (0.014) | 5.9  (0.055) | **0.153** | 807 | 5.1  (0.035) | 54.8  (0.239) | **0.931** |
| Occipital Lobe | 559 | 0.9  (0.017) | 6.1  (0.069) | **0.148** | 496 | 5.0  (0.06) | 55.2  (0.388) | **0.901** |
| Temporal Lobe | 492 | 0.9  (0.02) | 6.1  (0.08) | **0.148** | 368 | 4.8  (0.052) | 54.3  (0.359) | **0.088** |

A: The Ka, Ks, and their standard error (S.E. in parentheses) have been multiplied by 10^2^
