# Supplementary material for: Rate of Evolution in Brain-Expressed Genes in Humans and Other Primates
Source: PLoS Biol. 2006 Dec 26;5(2):e13. doi: 10.1371/journal.pbio.0050013 (PMC1717015; doi:10.1371/journal.pbio.0050013)
Supplement: Supplementary file 6 [file pbio.0050013.st003.doc]

Table S3. Ka and Ks values of brain-specific genes in the Human, Chimpanzee and OWM lineages as shown in Fig. 2B.

|  |  | Human | Chimpan-zee | OWM |
| --- | --- | --- | --- | --- |
| Brain-specific genes(262) | Ka  (A) | 0.00080  (134) | 0.00069  (115) | 0.00626  (1048) |
|  | Ks  (S) | 0.00671  (396) | 0.00587  (356) | 0.06042  (3563) |
|  | [Ka/Ks]_B_ | 0.119 | 0.117 | 0.104 |
| [Ka/Ks]_B_/[Ka/Ks]_G_  P value (vs. human) | | (54.6%)  -- | (55.2%)  0.45 | (60.6%)  0.16 |

The information of tissue specificity were originally from the Web site [http://www.hugeindex.org](http://www.hugeindex.org/) and used by previous study [12]. In this study, our sequenced brain cDNAs cover only 262 brain specific genes.
