# Supplementary material for: Rate of Evolution in Brain-Expressed Genes in Humans and Other Primates
Source: PLoS Biol. 2006 Dec 26;5(2):e13. doi: 10.1371/journal.pbio.0050013 (PMC1717015; doi:10.1371/journal.pbio.0050013)
Supplement: Supplementary file 7 [file pbio.0050013.st004.doc]

Table S4. The level of nucleotide divergence (in %) in the 5’-region of genes with greater than 20% or 40% expression difference between human and chimpanzee. The average 5’-region divergence for all genes in the genome is given for comparison.

| All genes | Brain-expressed genes | Liver-expressed genes |
| --- | --- | --- |
|  | **1.11**  n=6791 | **1.14**  n=5805 |
|  | Genes with > 20% expression difference between human and chimpanzee | |
| **1.12**  n=7802 | **1.13**  n=462 | **1.20*****  n=851 |
|  | Genes with > 40% expression difference between human and chimpanzee | |
|  | **1.16**  n=101 | **1.23****  n=269 |

** P < 0.01; *** P < 0.001 in the comparisons between liver-expressed genes and “all genes” (see Supplement for test details)
